# Metagenome-assembled genomes (MAGs) of the emerging pathogen *Shewanella algae* from enrichment of coastal seawater, sediment, and algae at St. John’s Island, Singapore

**DOI:** 10.1128/mra.00846-24

**Published:** 2025-01-29

**Authors:** Jia Yee Ho, Dalong Hu, Rebecca J. Case, Yann F. Boucher

**Affiliations:** 1 Singapore Centre for Environmental Life Sciences Engineering (SCELSE), National University of Singapore, , Singapore; 2 Saw Swee Hock School of Public Health, National University of Singapore and National University Health System, , Singapore; 3 Singapore Centre for Environmental Life Sciences Engineering (SCELSE), Nanyang Technological University, , Singapore; 4 Department of Biological Sciences, Nanyang Technological University, , Singapore; 5 Infectious Diseases Translational Research Program, Department of Microbiology and Immunology, Yong Loo Lin School of Medicine, National University of Singapore and National University Health System, , Singapore; University of Notre Dame, Notre Dame, Indiana, USA

**Keywords:** metagenome-assembled genome, *Shewanella algae*, culture-enriched metagenomics, environmental samples, Singapore

## Abstract

Coastal water, sediment, and algae samples were collected from St. John’s Island, Singapore, and enriched in either broth or agar. Metagenomic sequencing was carried out on DNA from these enrichments and analyzed. A total of 29 metagenome-assembled genomes had been successfully asserted to be a close representation of *Shewanella algae*.

## ANNOUNCEMENT

*Shewanella* spp. is an Alteromonadales bacterium that can be ubiquitously found in soil, freshwater, and marine environments. Recently, there has been an increasing trend of human infections and antibiotic resistance associated with *Shewanella algae* being reported (1–5). These range from chronic otitis and infection of skin and soft tissue to bacteremia (6). Water, sediment, and algae samples were collected from six different locations around St. John’s Island, Singapore (August 2022). The samples were enriched overnight at 37°C in either broth, MacConkey, buffered peptone water, and tryptic soy broth, or on agar, MacConkey, Pseudomonas, and tryptic soy agar. All bacterial colonies grown on the agar after overnight incubation were harvested by scraping them into 1.5 mL of brain-heart infusion broth, and 0.5 mL was used for DNA extraction. For broth enrichments, 1.8 mL of the cell suspension was aliquoted from the broth after overnight incubation and used for DNA extraction (QIAamp PowerFecal Pro DNA Kit). The sequencing library was prepared using Illumina TruSeq Nano DNA with dual barcoded TruSeq DNA UD Indexes. The library was then sequenced on Illumina HiSeqX version 2.5, 150 × 150 bp. A total of 98 samples were sequenced, and a total of 3 billion reads were generated, with an average of 20–40 million reads per sample.

For the recovery of metagenome-assembled genomes (MAGs), the Kneaddata pipeline version 0.7.7 (https://github.com/biobakery/kneaddata) was first used to remove low-quality reads, and the initial assembly was then generated using metaSPAdes version 3.15.5 (7). The metaWRAP pipeline version 1.3 (8) was then used for reads’ binning, with the threshold for bins set to >50% completeness and <10% contamination rate. The bins were then annotated using Prokka version 1.14.5 (9). The Genome Taxonomy Database and associated taxonomic classification toolkit (10) were used for a more accurate taxonomic identification, where the classify workflow was utilized. The core genome was identified utilizing Roary version 3.11.2 (11). RAxML-NG (12) was used to build the maximum likelihood tree under the GTR + G model with 1,000 bootstrap replicates. The detection of antimicrobial resistance (AMR) genes in the MAGs was done using Staramr (13). The Pathosystems Resource Integration Center (PATRIC) was utilized to detect virulence factors (14).

**Fig 1:**
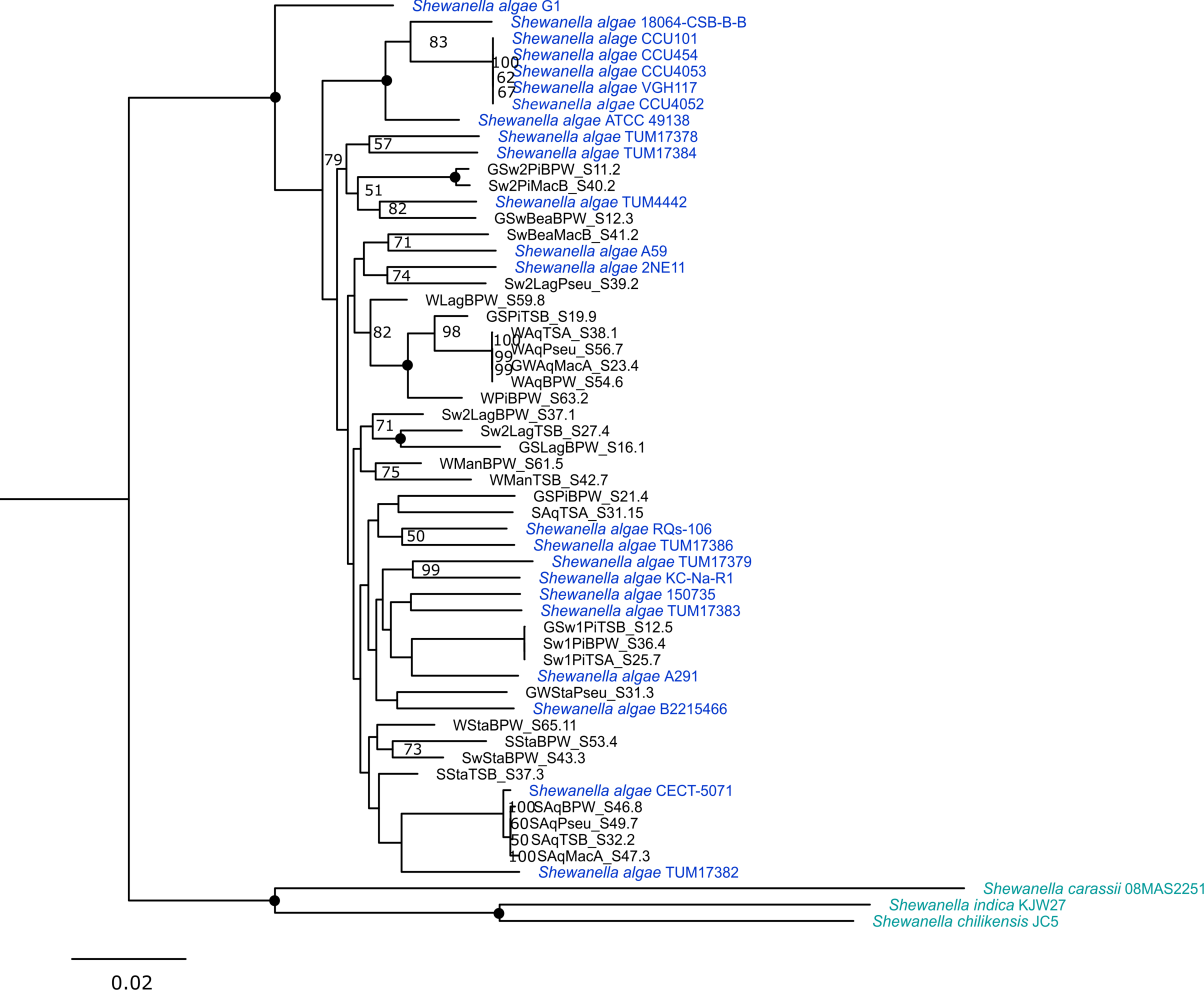
Maximum-likelihood tree of 987 core genes among 26 *Shewanella* spp. Blue text denotes *Shewanella algae* type strains, cyan text denotes outgroup of *Shewanella* spp. (*Shewanella carassii*, *Shewanella indica,* and *Shewanella chilikensis*), and black text denotes our MAGs. The scale bar indicates 0.02 nucleotide substitution per site. Nodes denote 100% bootstrap (Felsenstein’s bootstrap).

Thirty-one MAGs were identified as *S. algae*, of which 29 met the SeqCode standard (Table 1) (15). Their sizes and GC content are consistent with known *S. algae* characteristics (16, 17). A maximum-likelihood tree was reconstructed using 987 core genes from representative *S. algae* genomes (*n* = 23) and its close relatives (*n* = 3). The MAGs clustered with *S. algae* reference genomes, confirming their identification (Fig. 1). AMR genes detected in our MAGs are displayed in Table 1. Virulence factor genes curated in PATRIC for *S. algae* were screened using BLASTN (18). Notably, all MAGs carried OmpR, a known regulator for flagellum expression, biofilm formation, and virulence, as well as type VI secretion system (T6SS), including *hcp*, suggesting T6SS as a potential virulence factor in *S. algae* (19, 20).

**TABLE 1:** Genome features of the metagenome-assembled genomes asserted as *Shewanella algae*

MAGs (DDBJ BioSample number)	Completion (%)	Contamination (%)	G + C content (%)	Genome size (bp)	*N*_50_ (bp)	No. of contigs > 300 bp (accession number)	No. of CDSs^*a*^	No. of tRNA	No. of rRNA	AMR genes	Predicted AMR phenotype
GSLagBPW_S16 (SAMD00769434)	96.72	0.72	53	4,992,910	93,949	193 (BAAFPU010000001–BAAFPU010000193)	4,365	59	4	blaOXA-SHE, qnrA3	Ampicillin, ciprofloxacin
GSPiBPW_S21 (SAMD00769435)	97.37	0.54	53.1	4,684,716	92,622	104 (BAAFPV010000001–BAAFPV010000104)	4,097	55	1	blaOXA-SHE, qnrA3	Ampicillin, ciprofloxacin
GSPiTSB_S19 (SAMD00769436)	96.83	0.888	53.6	4,421,557	30,049	256 (BAAFPW010000001–BAAFPW010000256)	4,020	44	2	blaOXA-55, qnrA3	Ampicillin, ciprofloxacin
GSw1PiTSB_S12 (SAMD00769437)	96.91	0.694	53.1	4,664,281	71,950	121 (BAAFPX010000001–BAAFPX010000121)	4,120	47	3	blaOXA-SHE, qnrA4	Ampicillin, ciprofloxacin
GSw2PiBPW_S11 (SAMD00769460)	100	0.617	53.2	4,691,866	110,785	94 (BAAFQX010000001– BAAFQX010000094)	4,145	68	2	qnrA3	Ciprofloxacin
GSwBeaBPW_S12 (SAMD00769461)	98.42	0.617	53.1	4,781,714	94,943	120 (BAAFQY010000001–BAAFQY010000120)	4,181	88	3	mcr-4.3	Colistin
GWAqMacA_S23 (SAMD00769438)	98.03	1.853	53	4,833,651	94,937	112 (BAAFPY010000001–BAAFPY010000112)	4,243	63	2	blaOXA-55, qnrA7	Ampicillin, ciprofloxacin
GWStaPseu_S31 (SAMD00769462)	98.82	1.428	53.2	4,681,258	50,800	162 (BAAFQZ010000001–BAAFQZ010000162)	4,188	50	1	blaOXA-SHE, qnrA4	Ampicillin, ciprofloxacin
SAqBPW_S46 (SAMD00769439)	98	0.54	53.1	4,849,128	73,622	125 (BAAFPZ010000001–BAAFPZ010000125)	4,337	68	2	blaOXA-SHE, qnrA3	Ampicillin, ciprofloxacin
SAqMacA_S47 (SAMD00769440)	95.21	0.888	53.3	4,487,869	26,645	284 (BAAFQA010000001–BAAFQA010000284)	4,013	44	0	blaOXA-SHE, qnrA3	Ampicillin, ciprofloxacin
SAqPseu_S49 (SAMD00769441)	93.96	0.617	53.4	4,427,647	63,853	134 (BAAFQB010000001–BAAFQB010000134)	3,902	54	1	blaOXA-SHE, qnrA3	Ampicillin, ciprofloxacin
SAqTSA_S31 (SAMD00769442)	96.29	1.081	53.5	4,556,579	56,337	176 (BAAFQC010000001–BAAFQC010000176)	4,059	55	1	blaOXA-SHE, qnrA3	Ampicillin, ciprofloxacin
SAqTSB_S32 (SAMD00769443)	100	0.617	53.1	4,814,062	53,692	142 (BAAFQD010000001–BAAFQD010000142)	4,277	46	0	blaOXA-SHE, qnrA3	Ampicillin, ciprofloxacin
SStaBPW_S53 (SAMD00769444)	96.47	0.735	53.4	4,449,683	61,882	158 (BAAFQE010000001–BAAFQE010000158)	3,983	57	2	blaOXA-SHE, qnrA3	Ampicillin, ciprofloxacin
SStaTSB_S37 (SAMD00769445)	99.43	1.158	53.4	4,778,220	58,976	210 (BAAFQF010000001–BAAFQF010000210)	4,320	57	3	blaOXA-SHE, qnrA3	Ampicillin, ciprofloxacin
Sw1PiBPW_S36 (SAMD00769446)	100	0.617	53.1	4,765,173	170,219	66 (BAAFQG010000001–BAAFQG010000066)	4,148	88	3	blaOXA-SHE, qnrA4	Ampicillin, ciprofloxacin
Sw1PiTSA_S25 (SAMD00769447)	97.37	0.54	53.1	4,735,696	188,601	76 (BAAFQH010000001–BAAFQH010000076)	4,123	68	4	blaOXA-SHE, qnrA4	Ampicillin, ciprofloxacin
Sw2LagBPW_S37 (SAMD00769448)	99.72	0.617	53.6	4,542,580	36,415	195 (BAAFQI010000001–BAAFQI010000195)	4,022	46	1	blaOXA-SHE, qnrA3	Ampicillin, ciprofloxacin
Sw2LagPseu_S39 (SAMD00769449)	96.57	1.954	53.5	4,385,340	21,534	425 (BAAFQJ010000001–BAAFQJ010000425)	4,035	43	1	qnrA4	Ciprofloxacin
Sw2LagTSB_S27 (SAMD00769450)	90.74	1.338	53.6	4,337,359	8,574	681 (BAAFQK010000001–BAAFQK010000681)	4,265	28	0	blaOXA-SHE, qnrA3	Ampicillin, ciprofloxacin
Sw2PiMacB_S40 (SAMD00769451)	92.16	0.873	53.3	4,414,790	17,925	423 (BAAFQL010000001–BAAFQL010000423)	4,077	38	2	blaOXA-SHE, qnrA3	Ampicillin, ciprofloxacin
SwBeaMacB_S41 (SAMD00769452)	96.83	0.54	53.5	4,496,375	35,776	231 (BAAFQM010000001–BAAFQM010000231)	4,056	46	1	qnrA3	Ciprofloxacin
SwStaBPW_S43 (SAMD00769453)	99.18	0.617	53.5	4,563,608	41,985	202 (BAAFQN010000001–BAAFQN010000202)	4,055	66	4	blaOXA-SHE, qnrA3	Ampicillin, ciprofloxacin
WAqBPW_S54 (SAMD00769454)	100	0.617	53	4,773,274	134,150	57 (BAAFQR010000001–BAAFQR010000057)	4,168	60	2	blaOXA-55, qnrA7	Ampicillin, ciprofloxacin
WAqPseu_S56 (SAMD00769455)	96.83	0.54	52.9	4,858,153	170,597	58 (BAAFQS010000001–BAAFQS010000058)	4,243	62	4	blaOXA-55, qnrA7	Ampicillin, ciprofloxacin
WAqTSA_S38 (SAMD00769456)	98.91	1.698	53	4,875,049	77,346	126 (BAAFQT010000001–BAAFQT010000126)	4,276	75	8	blaOXA-55, qnrA7	Ampicillin, ciprofloxacin
WLagBPW_S59 (SAMD00769457)	98.55	0.617	53.7	4,623,295	49,923	202 (BAAFQU010000001–BAAFQU010000202)	4,100	50	2	qnrA3	Ciprofloxacin
WManBPW_S61 (SAMD00769458)	95.75	1.158	53.6	4,414,174	29,454	318 (BAAFQV010000001–BAAFQV010000318)	3,978	44	0	qnrA3	Ciprofloxacin
WStaBPW_S65 (SAMD00769459)	97.01	0.99	53.7	4,480,059	41,561	225 (BAAFQW010000001–BAAFQW010000225)	4,015	51	1	blaOXA-SHE, qnrA7	Ampicillin, ciprofloxacin

^*a*^ CDS, coding DNA sequences.

This study demonstrates the utility of culture-enriched metagenomics for generating high-quality MAGs of human pathogens found at low abundance in the environment.

## Data Availability

This metagenomics sequencing project has been deposited in the DDBJ Sequence Read Archive (DRA) under BioProject PRJDB17097. The raw reads used for assembling the MAGs are associated with BioSample numbers SAMD00659507–SAMD00659561 and SAMD00731969–SAMD00731994. The 29 MAGs are available under DDBJ accession numbers BAAFPU010000000–BAAFQZ010000000, with corresponding BioSample numbers listed in Table 1.
